# Novel DNA Bis-Intercalator XR5944 as a Potent Anticancer Drug—Design and Mechanism of Action

**DOI:** 10.3390/molecules26144132

**Published:** 2021-07-07

**Authors:** Adam J. Buric, Jonathan Dickerhoff, Danzhou Yang

**Affiliations:** 1 College of Pharmacy, Medicinal Chemistry and Molecular Pharmacology, 575 W Stadium Ave, Purdue University, West Lafayette, IN 47907, USA; aburic@purdue.edu (A.J.B.); dickerho@purdue.edu (J.D.); 2 Center for Cancer Research, Purdue University, 201 S University St, West Lafayette, IN 47906, USA; 3 Department of Chemistry, Purdue University, West Lafayette, IN 47906, USA; 4 Purdue Institute for Drug Discovery, West Lafayette, IN 47906, USA

**Keywords:** XR5944, MLN944, anticancer drug, DNA, NMR, bis-intercalator, major groove binder

## Abstract

This review is dedicated to Professor William A. Denny’s discovery of XR5944 (also known as MLN944). XR5944 is a DNA-targeted agent with exceptionally potent antitumor activity and a novel DNA binding mode, bis-intercalation and major groove binding, as well as a novel mechanism of action, transcription inhibition. This novel anticancer compound represents a remarkable accomplishment resulting from two decades of drug discovery by Professor Denny and coworkers. Here, we review our work on the structural study of the DNA binding mode of XR5944 and mechanistic study of XR5944 action.

## 1. Introduction

Despite tremendous advances in cancer therapeutics in recent decades, there is an obvious and urgent need for more effective anticancer drugs. DNA-targeted chemotherapeutics have been widely used and have substantially increased patient survival rates [1]. However, double-stranded (ds) DNA-interactive anticancer drugs are often accompanied by adverse effects at therapeutic dosages [2]. Therefore, anticancer drugs with different mechanisms of action are often used in combination therapy to enhance efficacy at reduced dosages, as well as to decrease the likelihood of acquired drug resistance [3]. Accordingly, significant effort has been put towards the development of new anticancer drugs with novel mechanisms of action.

**Figure 1:**
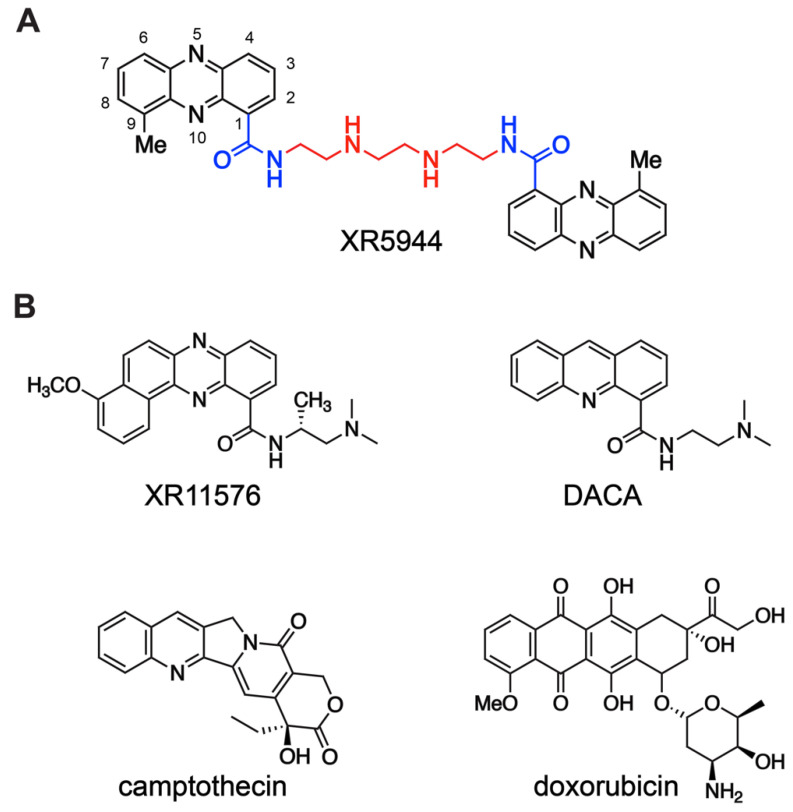
(**A**) Chemical structure of XR5944, bis(9-methylphenazine-1-carboxamide). Carboxamide substituents (blue) of each 9-methylphenazine chromophore (black) are coupled via a diamine linker (red). (**B**) Chemical structures of related monomer compounds XR11576 and DACA (top) and topoisomerase I inhibitor camptothecin and topoisomerase II inhibitor doxorubicin (bottom).

XR5944 (also known as MLN944), bis(9-methylphenazine-1-carboxamide) (Figure 1A) is an exceptionally potent antitumor agent with a novel DNA binding mode and mechanism of action, namely dsDNA bis-intercalation with major groove binding and transcription inhibition [4,5,6]. It displays exceptional cytotoxic potency against a wide range of human cancer cell lines, including leukemia and solid tumors such as colon, small cell lung carcinoma (SCLC), and non-small cell lung carcinoma, with in vitro EC50 in the range 0.04–0.4 nM [7,8]. It also is highly active against multidrug resistant human cancer cells. Furthermore, XR5944 demonstrates antitumor efficacy in human xenograft models in vivo, including the H69 SCLC model and the chemorefractory HT29 colon carcinoma model. Further study showed XR5944 exhibits potent ex vivo sensitivity to a variety of solid tumors, particularly breast and ovarian carcinoma [9]. Additionally, XR5944 shows significant activity in combination with several clinical antitumor agents in human carcinoma cell lines and xenografts [10,11]. Based on the promising preclinical data, XR5944 entered a phase I clinical trial for solid tumors in 2003 [12].

The novel bisphenazine compound XR5944 represents a remarkable accomplishment resulting from two decades of drug design and development by William Denny and coworkers. Early research by Denny et al. focused on developing structure–activity relationships (SAR) and optimization of acridine carboxamides [13,14] and phenazine carboxamides [15] (Figure 1B top) as dual DNA topoisomerase I and II inhibitors. A planar tricyclic chromophore that intercalates dsDNA and a ring-nitrogen peri to the carboxamide are required for antitumor activity [16]. Chromophores bearing low pK_a_ ring nitrogens, such as acridine-4-carboxamide (pK_a_ = 3.54) [14] and phenazine-1-carboxamide (pK_a_ = 0.84) [16], are uncharged at physiological pH and thus more lipophilic, which is correlated with more effective tumor distribution in vivo. A series of dimeric analogues of lipophilic, functionally neutral tricyclic carboxamides was prepared. Remarkably, the dimerization of tricyclic aromatic carboxamides significantly enhanced their antitumor potency compared to the corresponding monomers in a panel of cell lines [17]. Particularly, the dimeric analog of phenazine-1-carboxamide showed the greatest increase in cytotoxicity compared to its monomeric counterpart [17]. The addition of a methyl group peri to the ring N10 of the phenazine chromophore improved anticancer activity [18]. Further linker chain SAR revealed that the dicationic—(CH_2_)_2_NH(CH_2_)_2_NH(CH_2_)_2_—linker yielded the greatest cytotoxicity of bis-intercalators [8]. Altogether, these led to the discovery of XR5944 (Figure 1A), which shows exceptional antitumor cytotoxic potency.

The parent monomeric phenazine carboxamides of XR5944 and the closely related acridine carboxamides act via dual topoisomerase I/II inhibition, such as phenazine carboxamide XR11576 [19] and acridine carboxamide DACA (Figure 1B top) [20]. However, the bisphenazine compound XR5944 showed significantly higher in vitro and in vivo potency than the traditional topoisomerase I inhibitor camptothecin derivatives or topoisomerase II inhibitor doxorubicin (Figure 1B) [7,9]. Although initial testing suggested that XR5944 may bind DNA and inhibit topoisomerase activity [8], later studies revealed a cytotoxic mechanism primarily independent of topoisomerase inhibition [21]. XR5944 does not notably increase DNA cleavage mediated by either topoisomerase I or II at its cytotoxic concentration. Type I and type II topoisomerase knockdown yeast mutants had no effect on XR5944 cytotoxicity. Furthermore, many genes were regulated differentially upon treatment with XR5944 versus the known topoisomerase I inhibitor camptothecin derivative irinotecan [21]. Intriguingly, the mechanism of cytotoxicity of XR5944 was subsequently determined to be transcription inhibition [5].

Here, we review the work in our lab on structural studies of the DNA binding mode of XR5944 and mechanistic studies of XR5944 action. Our study shows that XR5944 bis-intercalates dsDNA, with its diamine linker positioned in the major groove, and inhibits the DNA binding of transcription factor estrogen receptor (ER) to the estrogen response element (ERE) in gene promoters. Our structural and mechanistic study elucidates the molecular mechanisms of transcription inhibition of XR5944 and provides information for the rational design of bis-intercalating anticancer drugs targeting ERE DNA for ER inhibition.

## 2. NMR Solution Structure of XR5944 in Complex with the d(ATGCAT)_2_ Duplex DNA

**Figure 2:**
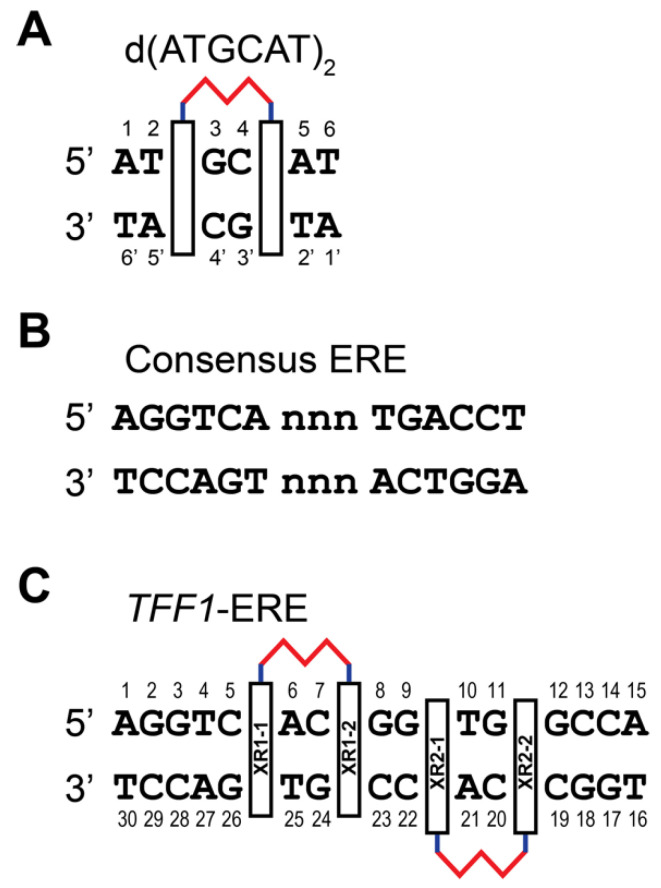
(**A**) XR5944 preferred binding site of palindromic DNA sequence d(ATGCAT)_2_. Two chromophores intercalate at a 5′-TpG / 5′-CpA site, enveloping two base pairs. (**B**) Consensus ERE sequence with variable “nnn” trinucleotide spacer. (**C**) Two binding sites of XR5944 to the naturally occurring TFF1-ERE DNA sequence.

**Figure 3:**
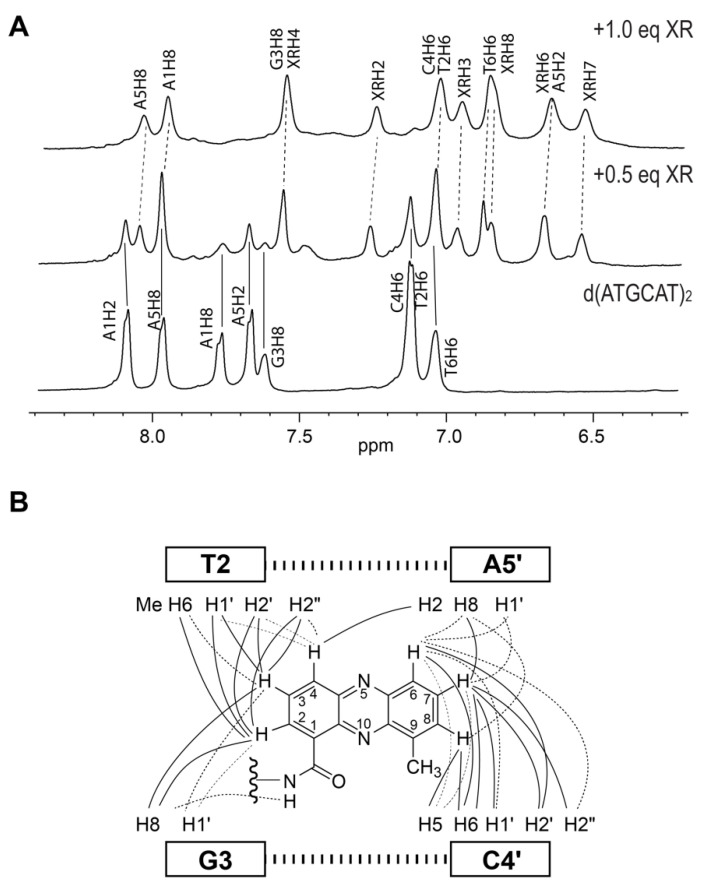
(**A**) The aromatic proton region of 1D ^1^H NMR titration of XR5944 to dsDNA d(ATGCAT)_2_. Titration of XR5944 diminishes the peaks of the free DNA (labeled by solid lines), accompanied by the emergence of a new set of well-resolved peaks of the drug–DNA complex (dashed lines). (**B**) Schematic diagram of the intermolecular NOE interactions between XR5944 and DNA. Strong, medium and weak NOE interactions are indicated by bold solid lines, solid lines and dashed lines, respectively.

We first determined the preferred DNA binding site of XR5944 and its DNA binding mode [4]. Knowing the DNA binding of XR5944 at the atomic level can provide insights into its mechanism of action and can guide structure-based rational drug design. XR5944 was screened against a range of palindromic duplex DNA sequences using 1D ^1^H NMR titration. The best binding sequence was shown to be d(ATGCAT)_2_ (Figure 2A). The appearance of a single set of new well-resolved proton resonances at a 1:1 drug:DNA ratio indicated the formation of a well-defined XR5944-DNA complex with two-fold symmetry (Figure 3A). Excellent NMR spectral quality of the complex showed duplex d(ATGCAT)_2_ to be an ideal sequence for NMR structure determination of the XR5944-DNA complex. Moreover, 2D NOESY experiments of the XR5944-DNA complex revealed intermolecular interactions that clearly located the intercalating phenazine moieties of the drug between the T2pG3:(C4′pA5′) and C4pA5:(T2′pG3′) base pairs (Figure 2A and Figure 3B).

**Figure 4:**
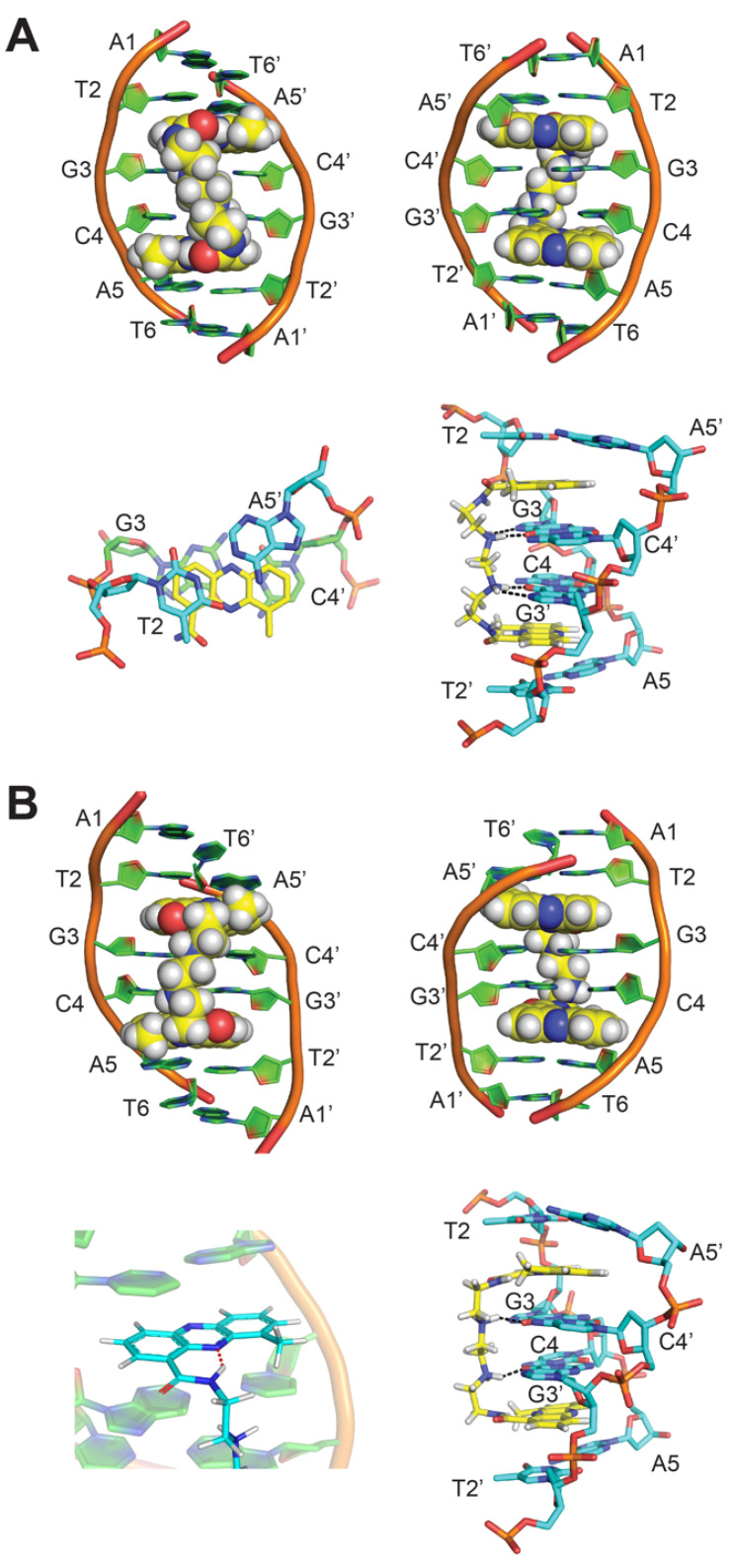
(**A**) NMR solution structure of the 1:1 complex of XR5944 to palindromic d(ATGCAT)_2_ [PDB: 1X95]: (top) the complex structure as viewed from major groove (left) and minor groove (right). (bottom left) Intercalation mode of XR5944 phenazine chromophore (yellow) at the 5′-TpG sites. Carbon atoms of DNA bases enveloped by bis-intercalation are colored green. Carbon atoms of DNA bases external to bis-intercalation are colored cyan. (bottom right) Major groove hydrogen bond interactions of the linker shown in black dashes. (**B**) Updated NMR solution structure of 1:1 complex of XR5944 to d(ATGCAT)_2_ [PDB: 4BZT]. (top) The complex structure as viewed from major groove (left) and minor groove (right). (bottom left) XR5944 intramolecular hydrogen bond formed between N10 and carboxamide N-H. (bottom right) Major groove hydrogen bond interactions of the linker shown in black dashes.

We determined the NMR solution structure of the 1:1 complex of XR5944 to palindromic duplex DNA d(ATGCAT)_2_, which provided a molecular basis for the specific DNA recognition of XR5944 at the preferred 5′-TGCA binding site (Figure 4A) [4]. XR5944 bis-intercalates at the two symmetric T_2_pG_3_:(C_4′_pA_5′_) and C_4_pA_5_:(T_2′_pG_3′_) sites, with its two phenazine chromophores flanking the two central G-C base pairs (Figure 4A). The drug dicationic diamine linker is located in the DNA major groove (Figure 4A top) and hydrogen-bonded with the two guanine bases on the opposite strands (Figure 4A bottom right). The aromatic phenazine moieties adopt a parallel-intercalation mode, with their long axes parallel to the long axes of the intercalated base pairs (Figure 4A bottom left).

More recently, an updated structure of XR5944 bound to the same d(ATGCAT)_2_ sequence was reported (Figure 4B), for which the N10 of the phenazine ring was identified as unprotonated [22]. An intramolecular hydrogen bond can be formed between the N-H of the carboxamide and N10 of the phenazine ring (Figure 4B bottom left). This hydrogen bond leads to a reversed conformation of the carboxamide moiety and a right-handed twist conformation of the linker in the major groove (Figure 4B top).

The NMR structure of the XR5944-d(ATGCAT)_2_ duplex DNA complex reveals that XR5944 bis-intercalates preferably at the 5′-TGCA sites, with its long axis parallel to the Watson–Crick hydrogen bonds, similar to the binding mode of the acridine carboxamide 9-amino-DACA [23,24]. XR5944 wraps two base pairs, with its diamine linker positioned in the major groove of the duplex DNA [4]. Whereas most groove-binding small molecules bind to the DNA minor groove [25], transcription factors and other DNA-binding proteins typically interact with the major groove of duplex DNA [26]. The occupancy of the major groove by the diamine linker is thereby capable of disrupting the major groove interactions required for most transcription factor bindings. Therefore, the structures of XR5944 in complex with duplex DNA provide molecular-level details of its DNA binding mode and insights into its mechanism of action of transcriptional inhibition.

## 3. XR5944 Binds the ERE Sequence and Inhibits ERα-ERE Interaction

**Figure 5:**
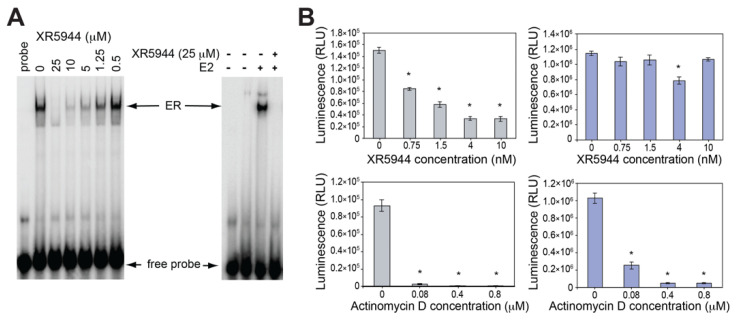
(**A**) Electrophoretic mobility shift assays (EMSA) showing that XR5944 inhibits the ER binding to consensus ERE DNA probe using ER extracted from MCF-7 nucleus (left) and recombinant ERα in the presence of estrogen (right). (**B**) XR5944 decreases the ERE-promoted transcription (left) but not the pGL3-promoted transcription (right) in luciferase reporter assay. The general transcription inhibitor actinomycin D decreases both transcriptions (bottom). *Columns*, mean of three independent experiments; *bars*, standard error; * *p* < 0.01, statistically significant difference. Modified from Punchihewa, C.; De Alba, A.; Sidell, N.; Yang, D. XR5944: A Potent Inhibitor of Estrogen Receptors. Mol. Cancer Ther. 2007, 6 (1), 213–219.

The preferred DNA binding site 5′-CpA of XR5944 is part of the estrogen response element (ERE) consensus sequence (Figure 2B), the DNA-binding site of transcription factor estrogen receptor-α (ERα). This ligand-activated transcription factor is the predominant mediator of estrogen response in breast cancers [27]. ERα binds the ERE promoter sequence and promotes downstream transcription [28]. We examined the ability of XR5944 to inhibit ER activity in vitro and in cultured cells [29]. XR5944 inhibits ERα protein binding to ERE DNA in a dose dependent manner (Figure 5A), as shown in an electrophoretic mobility shift assay. Luciferase reporter assays revealed that XR5944 specifically inhibits ERE-promoter dependent gene expression, but does not affect a basal promoter (Figure 5B) or Sp1 or NF-kB transactivation [29]. Therefore, XR5944 is able to specifically inhibit ERα binding to its ERE sequence in vitro and inhibits ERα transactivation in vivo.

**Figure 6:**
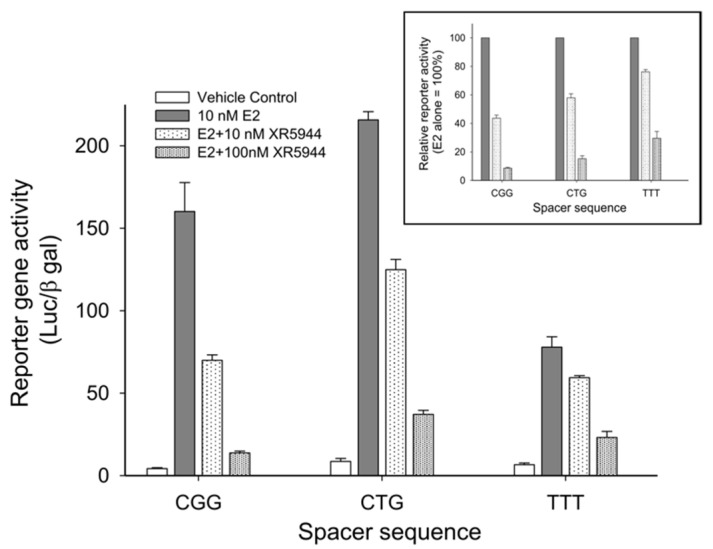
Effect of trinucleotide spacer sequence on XR5944 downregulation of ERE-promoted genes. XR5944 showed greatest suppression of CGG-containing ERE promoters, followed by CTG and TTT spacers, respectively. Copy from Figure 4A, Sidell 2011. This article was published in J. Steroid Biochem. Mol. Biol. 2011, 124 (3–5), Sidell, N.; Mathad, R.I.; Shu, F.; Zhang, Z.; Kallen, C.B.; Yang, D. Intercalation of XR5944 with the Estrogen Response Element Is Modulated by the Tri-Nucleotide Spacer Sequence between Half-Sites. p121–127. Copyright Elsevier (2011).

Consensus ERE sequences consist of two conserved half-sites spaced by a variable trinucleotide spacer (Figure 2B). We examined the effect of different trinucleotide spacers on XR5944 binding to the consensus ERE sequence in vitro [30]. Variation of the “nnn” tri-nucleotide spacer significantly affects XR5944 binding to ERE, with the best binding sequences containing the CGG spacer [30]. Likewise, the XR5944-induced transcriptional inhibition of ERE-promoter dependent genes is also modulated by the trinucleotide spacer sequence in vivo (Figure 6). Subsequently, we showed that the spacer sequence is conserved amongst ERα-bound ERE sequences [31].

These studies demonstrated that XR5944 binds to ERE DNA sequences and is a potent inhibitor of estrogen receptor activity. The drug binding to ERE sequences and inhibition of ERE-promoted gene expression is modulated by the trinucleotide spacer sequence between the two ERE half-sites [30]. This novel mechanism of action may prove valuable in overcoming drug resistance to currently marketed antiestrogen treatments, all of which target the hormone-receptor complex.

## 4. Solution Structure of 2:1 Complex of XR5944 with the Naturally Occurring ERE Sequence of *TFF1* Gene

**Figure 7:**
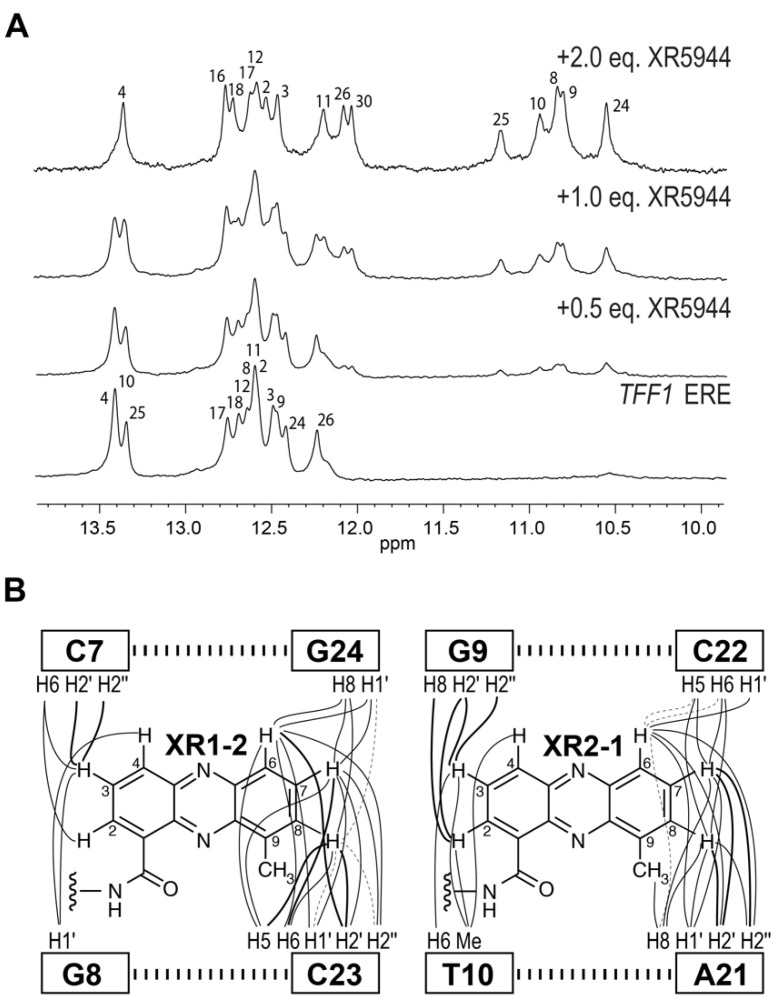
(**A**) The imino proton region of 1D ^1^H NMR titration of XR5944 with the naturally occurring *TFF1* ERE sequence, which contains a CGG tri-nucleotide spacer. Titration of XR5944 diminishes the imino proton peaks of the free DNA, accompanied by the emergence of a new set of well-resolved upfield peaks of the drug–DNA complex. (**B**) Schematic diagram of intermolecular NOEs between the two strong intercalating XR5944 moieties: XR1–2/XR2–1 and the TFF1 DNA protons. Strong, medium and weak NOE interactions are indicated by bold solid lines, solid lines and dashed lines, respectively.

To understand the molecular level interactions of XR5944 with the ERE DNA sequence, we decided to determine the molecular structure of XR5944-ERE DNA complex. We first screened the binding of XR5944 to various naturally occurring ERE sequences by 1D NMR titration experiments to identify the best binding sequence. XR5944 showed the highest binding specificity to the ERE sequences of *TFF1* (Trefoil factor1, previously known as *PS2*) (Figure 2C) and *TGF-α* genes, both containing a CGG spacer. The disappearance of unbound DNA imino proton resonances and the emergence of a single set of new upfield-shifted proton resonances at two equivalents of XR5944 indicated the formation of a single, well-defined XR5944-DNA complex with a 2:1 binding stoichiometry (Figure 7A) [30]. The complex shows good NMR spectral quality and we determined the NMR solution structure of the 2:1 complex of XR5944 bound to the naturally occurring ERE sequence of the *TFF1* gene [32]. The NOESY data of the complex revealed numerous intermolecular interactions that defined the location of two strong intercalation sites of the XR5944 molecule, with one being C7pG8:(G24pC23) and the other being G9pT10:(C22pA21) (Figure 7B).

**Figure 8:**
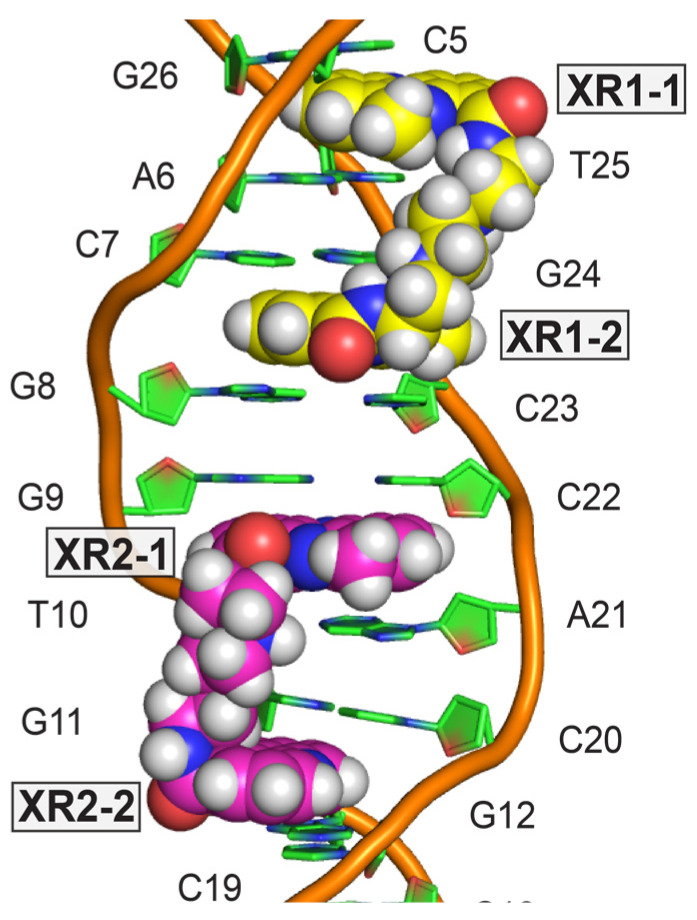
NMR solution structure of 2:1 complex of XR5944 with *TFF1*-ERE DNA [PDB: 2MG8]. Model representation of solved structure showing bis-intercalators XR1 (yellow) and XR2 (magenta).

Two XR5944 molecules bis-intercalate at adjacent sites within the *TFF1*-ERE DNA, both involving the trinucleotide spacer CGG (Figure 2C) [32]. Similar to the binding in the 1:1 complex with d(ATGCAT)_2_ [4], cationic diamine linkers of XR5944 molecules are positioned in the major groove, with each bis-intercalator enveloping the central two base-pairs (Figure 8). In both drug complexes, XR5944 binds strongly at one intercalation site but weakly at another. No intramolecular hydrogen bonds were observed between N10 and its respective carboxamide for XR5944 in the *TFF1*-ERE DNA complex. XR5944 appears to form hydrogen-bond interactions with guanine or thymine carbonyl groups in the DNA major groove. The flexibility of the carboxamide conformation appears to play an important role in the major-groove hydrogen-bond interactions with various DNA binding sequences.

## 5. Disrupting ERE-ERα Complex Formation: A Potential Alternative to Antiestrogen Therapeutics

Approximately two-thirds of all breast cancers express ERα [33]. Estrogen binds to, and activates, ERα. The activated hormone receptor binds to ERE and promotes transcription of downstream genes. As such, its overexpression can cause hyperactive cell proliferation and growth [34]. ER-expressing cancers are predominantly treated with endocrine therapy, such as antiestrogens that antagonize estrogen binding to ERα [35]. However, chronic administration of antiestrogen therapeutics can ultimately lead to antiestrogen resistance [35,36], in addition to de novo resistance to antiestrogen therapy. The molecular interactions of XR5944 with the *TFF1*-ERE duplex DNA show how XR5944 may inhibit ERE-ERα complex formation. The diamine linker of the XR5944 bis-intercalator blocks the major groove, potentially inhibiting essential major groove contacts required for transcription factor ERα binding. Disruption of the ER-ERE protein-DNA interactions by XR5944 binding to the duplex major groove is a novel method for inhibiting an ERα-mediated transcriptional response, which represents a potential means to bypass antiestrogen resistance in endocrine cancer therapeutics because ER-DNA binding is required for all antiestrogen-resistant estrogen-independent ERα receptor activations.

## 6. Conclusions

The bis(phenazinecarboxamide) XR5944 represents a unique class of DNA-binding small molecules. It binds to the major groove of a DNA duplex and is guided by bis-intercalation. XR5944 shows exceptional activity against a wide range of human tumors with a mechanism of transcription inhibition, and it further expands the potential of pharmacopeia to seek new targets and mechanisms of action. The NMR structures of XR5944 bound to duplex DNA provide the molecular basis of XR5944’s DNA recognition through a combination of bis-intercalation with the two phenazine chromophores and major groove interactions with the diamine linker. This novel DNA interaction mode provides insight into the mechanism of transcription inhibition by preventing the DNA major groove interactions of transcription factors. One such transcription factor is ERα. XR5944 specifically binds to the ERα cognate DNA binding site, ERE DNA sequences, to inhibit ER activity. This novel mechanism of action may prove valuable in overcoming drug resistance to currently marketed antiestrogen treatments, all of which target the hormone-receptor complex. The NMR structure of XR5944 bound to a native ERE sequence shows that binding involves the tri-nucleotide spacer. This structural information provides a molecular basis for the future structure-based rational design of bis-intercalators as ERE-targeted ER inhibitors, as well as DNA-targeted transcription inhibitors in general.
